# An automated method for the determination of deoxyribonuclease activity as exemplified by fractionation of the components of the medicament Varidase^®^

**DOI:** 10.1155/S1463924693000100

**Published:** 1993

**Authors:** I. C. Locke, M. P. Ramsey, S. S. Hill, B. G. Carpenter

**Affiliations:** 1 School of Pharmacy and Biomedical Sciences University of Portsmouth King Henry I Street Portsmouth PO1 2DZ UK; 2 Lederle Laboratories Cyanamid of Great Britain Ltd Fareham Road Gosport PO13 0AS UK; 3 Pharmaceutical Unit St Mary's Hospital Corbett Road South Glamorgan, Wales Penarth UK

## Abstract

The activity of most deoxyribonuclease enzymes can be monitored by measuring the change in absorbance at 260 nm which accompanies the breakdown of the double-stranded structure of native DNA. An automated method for determining deoxyribonuclease activity, based on such an absorbance change, which can overcome problems of inhibition arising from the presence of inorganic cations, is described. Variations in inorganic cation concentration is a particular problem when measuring the activity of chromatographic fractions eluted via a salt gradient. A comparison is made between the automated and a manual method for the assay of deoxyribonuclease active constituents, of the medicament ‘Varidase’, eluted from a Cellex-D (Bio-Rad Laboratories Ltd) anionic exchange resin using a 0.05-1.0 M sodium chloride gradient.


Journal of Automatic Chemistry, Vol. 15, No. 2 (March-April 1993), pp. 65-70

### An automated method for the determination of deoxyribonuclease activity as exemplified by fractionation of the components of the medicament Varidase®

**I. C. Locke, M. P. Ramsey*, S. S. Hill† and B. G. Carpenter**

*School of Pharmacy and Biomedical Sciences, University of Portsmouth, King Henry I Street, Portsmouth PO1 2DZ, UK*

*The activity of most deoxyribonuclease enzymes can be monitored by measuring the change in absorbance at 260 nm which accompanies the breakdown of the double-stranded structure of native DNA. An automated method for determining deoxyribonuclease activity, based on such an absorbance change, which can overcome problems of inhibition/activation arising from the presence of inorganic cations, is described. Variations in inorganic cation concentration is a particular problem when measuring the activity of chromatographic fractions eluted via a salt gradient. A comparison is made between the automated and a manual method for the assay of deoxyribonuclease active constituents, of the medicament 'Varidase', eluted from a Cellex-D (Bio-Rad Laboratories Ltd) anionic exchange resin using a 0·05–1·0 M sodium chloride gradient.*

### Introduction

Streptodornase-streptokinase Varidase® is a medicament derived from the refined extra-cellular products of a Lancefield group C strain of *Streptococcus* [1], where the principal therapeutically active constituents are streptokinase, a plasminogen activator, and a group of deoxyribonucleases collectively described as streptodornase. In the United Kingdom, Varidase is used topically for the treatment of inflammation and the degradation and liquefaction of accumulations of clotted blood and fibrinous and purulent exudates [2]. However, Varidase is also produced as an oral anti-inflammatory, which finds widespread use in a number of countries throughout the world.

The two principal active components of the medicament have separate, but mutually enhancing, roles. Streptokinase activates plasminogen to form plasmin, which causes the breakdown of fibrin resulting in a rapid dissolution of both blood clots and the fibrinous portion of the wound exudate [3]. The clumping of leucocytes, which prevents good wound drainage, is caused mainly through extracellular nucleoprotein. Degradation of the DNA, by the streptodornase, liquefies the pus, allowing the free movement of leucocytes and enhanced phagocytosis.

At a molecular level, there is a considerable understanding of the mode of action of streptokinase, but little is known of the mechanisms of degradation of DNA by streptodornase and the possible influence of cations and other constituent proteins. As a step towards such an understanding, the isolation of those protein constituents of Varidase which have deoxyribonuclease activity has been attempted.

The topical Varidase preparation contains 25–30% protein. SDS polyacrylamide gel electrophoretic analysis reveals well over 20 different species of protein [4]. Attempts to isolate those species which have deoxyribonuclease activity from the total Varidase mixture have focused on a number of chromatographic procedures. In these experiments the large bed volume necessary to obtain acceptable resolution often results in between 150–200, 5 ml, fractions being collected per experiment. Analysis of these profiles is a daunting task, especially when three to five similar chromatographic separations are required to obtain sufficient material for the characterization experiments of the nucleases.

One approach for the assay of deoxyribonuclease activity is to observe the change in absorbance at 260 nm which accompanies the breakdown of the DNA double-helical structure. The activity of the nuclease can then be expressed as the change in absorbance at 260 nm per unit absorbance per unit time. Consequently, the Technicon AutoAnalyser has been adapted to sample the separate column fractions and determine their corresponding nuclease activity automatically. The method, which introduces some novel features, and results from two different elution profiles is presented in this paper.

The elution conditions for some of the chromatographic procedures required a continually changing sodium chloride gradient. Increases in this salt are known to exert a considerable inhibitory influence on streptodornase activity [4–6]. These increases also apparently cause any DNA bound to glass tubing to desorb, hence the system had to be adapted for its removal.

### Experimental

### *Instrumentation*

An automated, continuous flow system was constructed based on the Technicon AutoAnalyser II (Technicon Instruments Corporation, Tarrytown, New York, USA)

* Lederle Laboratories, Cyanamid of Great Britain Ltd, Fareham Road, Gosport PO13 0AS, UK.
† Present address: Pharmaceutical Unit, St Mary's Hospital, Corbett Road, Penarth, South Glamorgan, Wales, UK.
® Registered Trademark.

0142-0453/93 $10.00 © 1993 Taylor & Francis Ltd.

consisting of the following units: Sampler IV, Proportioning pump III and Cartridge unit II (type A). The detector unit was a Perkin-Elmer 124 UV/Vis Spectrophotometer (Perkin-Elmer Ltd, Beaconsfield, UK).

Two systems were developed for use. The initial work, which did not involve solutions containing sodium chloride, was carried out on a basic schematic to which a dialysis section was added later (figure 1). The cartridge unit temperature was stabilized at 30 °C using a thermostat. Individual flow rates of each stream (shown in figure 1) were determined by the internal diameter of the tubing used as the Proportioning pump III rotates at a constant velocity. The dialysis units were fitted with Technicon Type C premount dialysis membranes (pore size 4–6 nm). Technicon part numbers are given in the schematic text to the specified component.

The sample stream, as delivered from the cups, was driven at a speed of 0·05 ml/min and mixed with the DNA substrate stream flowing at 2·5 ml/min. The absorbance at 260 nm was read at a constant time, approximately 3 min, after mixing using a 1 cm path length flow cell. The flow cell assembly incorporated a debubbler which removed all air bubbles before measurement.

In between processing of individual samples the Sampler IV probe underwent a wash sequence using a solution of 0·5 ml/l BRIJ-35 (30% solution) (Technicon Chemicals Company, ORCQ, Belgium), a non-ionic surfactant.

The dialysis was carried out against distilled water.

The manual assay used for comparison of the automatically determined activities was carried out using a Perkin-Elmer Lambda 5 UV/Vis Recording Spectrophotometer. The change in absorbance at 260 nm, after adding 50 μl of a column fraction to 2·5 ml of buffered DNA held in a 1 cm path length cuvette, was recorded over a 2·5 min time period. The activity was calculated from the initial (linear) slope of the recorded profile.

The assay data presented in this paper concern the analysis of two different chromatographic systems, gel filtration and ion-exchange. The gel filtration was carried out on a Bio-Gel P100 (Bio-Rad Laboratories Ltd, Hemel Hempstead, UK) column of 1200 ml volume using 0·05 M phosphate buffer (pH 7·4) and a sample loading of 300 mg. Ion-exchange was performed with Cellex-D anionic exchange resin (Bio-Rad Laboratories Ltd) using a column volume of 25 ml. The mobile phase was 0·05 M phosphate buffer (pH 7·4) containing varying concentrations of sodium chloride (a linear gradient between 0·05 M and 1·0 M).

*Reagents*

0·1 M magnesium sulphate, 0·1 M sodium hydroxide, 0·1 M sodium acetate (all Analar Merck) were prepared by dissolving the appropriate amount of the solid salt in distilled water. 1·0 M calcium chloride solution (AVS Merck) was prepared by diluting a stock 1·0 M solution.

Acetate buffer (pH 5·0) was prepared by mixing 1·0 M sodium acetate (20 ml), 0·1 M acetic acid (12·5 ml) and adding distilled water to a volume of 200 ml.

Phosphate buffer (pH 7·4) was prepared by mixing 0·1 M potassium dihydrogen phosphate (500 ml), 0·1 M sodium

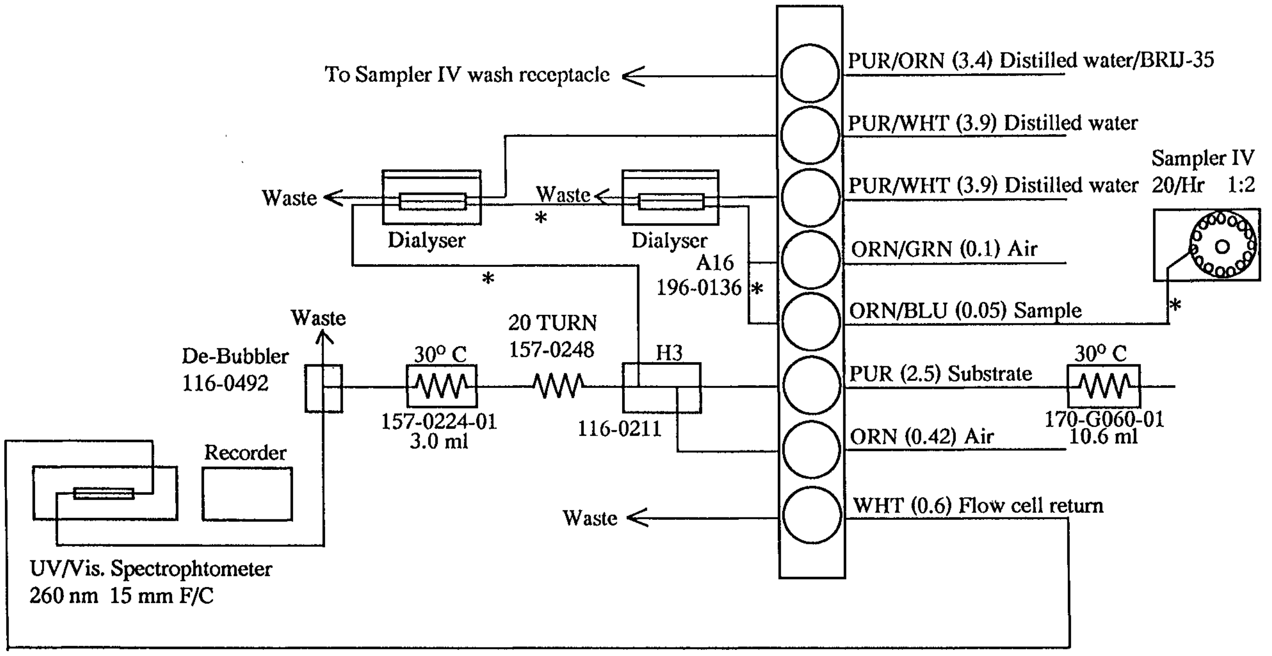

* denotes 116-0536-07 micro-bore tubing
All flow rates (quoted in brackets) are in ml/min.

*Figure 1. Schematic of AutoAnalyser components used. Details and Technicon part numbers are given next to the individual component where applicable. The schematic is drawn using standard AutoAnalyser notation for the components.*

hydroxide (393·4 ml) and adding distilled water to a volume of 1 l.

Deoxyribonucleic acid (DNA) substrate was prepared in Kunitz Buffer as follows: 40 mg of calf thymus DNA (highly polymerized, Sigma) was dissolved in approximately 200 ml of distilled water at room temperature. The following reagents were then added: 100 ml acetate buffer (pH 5·0); 42 ml magnesium sulphate (0·1 M); and 42 ml calcium chloride (0·1 M). The mixture was then made up to 1 l with distilled water and BRIJ-35 (30% solution) added at a concentration of 0·5 ml/l to facilitate the establishment of a good hydraulic pattern within the Autoanalyser system.

*Sample preparation*

No special preparation was necessary. Aliquots of the fractions collected from the chromatographic columns were either transferred into Sampler IV cups prior to autoanalysis, or injected directly into a cuvette for manual analysis.

### Results and discussion

The first consideration in developing the schematic for the Technicon AutoAnalyser was to achieve a spectroscopic change of sufficient magnitude, which could be monitored accurately and directly related to nuclease activity. The change in absorbance at 260 nm, resulting from the breakdown of the DNA double-helical structure at a fixed time of approximately 3 min after mixing fraction and substrate at 30 °C, proved most satisfactory. However, inorganic ions, and, in particular sodium chloride, are known to influence deoxyribonuclease activity. [7–10]. Thus, where chromatographic fractions result from elution with a sodium chloride gradient, varying degrees of inhibition would be expected with increasing salt concentration. An activity profile which did not compensate for the variation in salt would be criticized for not being a true representation.

To alleviate this problem, two dialysis units were employed in series (see figure 1), with separate distilled water supplies to each. These conditions were found to maximize the efficiency of the sample dialysis, giving a greater reduction in sodium chloride effects than either one dialysis unit alone, or two units with a linked distilled water supply.

As well as affecting the activity of nucleases, changing the sodium chloride levels had an important practical effect on the Autoanalyser system itself. When a sample containing an enhanced salt concentration entered the flow cell, there was a recorded increase in absorbance at 260 nm, even though the enhanced salt fraction did not contain any protein. False positives were being generated purely through the changes in salt concentration. Due to its viscous and polyanionic nature, the DNA substrate has a tendency to adhere to the walls of the vessels carrying it. It is possible that the observed increase in absorbance was the result of desorption of DNA, previously absorbed on the walls of the flow cell, brought about by an increase in salt. The increase in absorbance at 260 nm is not a property of the salt causing a change in the molar absorptivity of the DNA. Mixing 50 µl of a high salt concentration solution with 2·5 $cm^3$ of the DNA substrate solution results in a slight decrease due to the overall dilution effect.

The addition of the dialysis units, which minimized salt variations, resulted in a considerable decrease in absorption effects. However, in order to effect total removal of the false positives brought about by DNA adhesion, it was necessary to de-activate the flow cell. Several methods were investigated and found to be effective, but the improvement was short-lived, probably due to the coating being washed off by the continuous flow of liquid. Long term de-activation was achieved by the use of a proprietary water-repellent preparation: 'Repelcote' (Merck). This is a 2% solution of dimethyldichlorosilane in 1,1,1-trichloroethane, which, when used according to the manufacturer's instructions, forms a silicone film on the inside surface of the flow cell reducing the adhesion of aqueous solutions. This coating, in combination with the dialysis system, completely eliminated false positives.

The deoxyribonuclease assay gives an optimum response when performed at 30 °C. To obtain this temperature the reaction mixture was passed through a heating coil kept at that temperature in a water bath. It was found, however, that the amount of time spent in this heating coil was not sufficient to raise the temperature of the contents to 30 °C. As the length of this heating coil could not be extended without greatly increasing the incubation time, the problem was overcome by employing a second

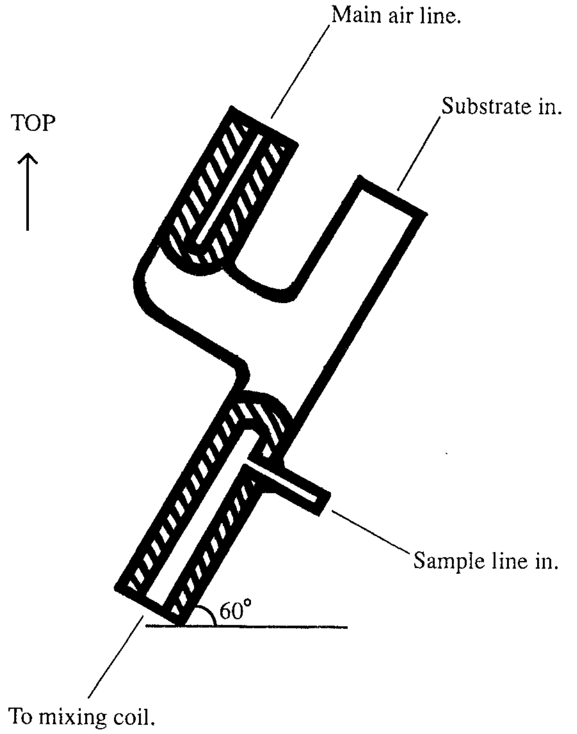

*Figure 2. Illustration to show the orientation of the Technicon 'h3' component used to promote the highest degree of bubble coalition. Angles quoted are approximate.*

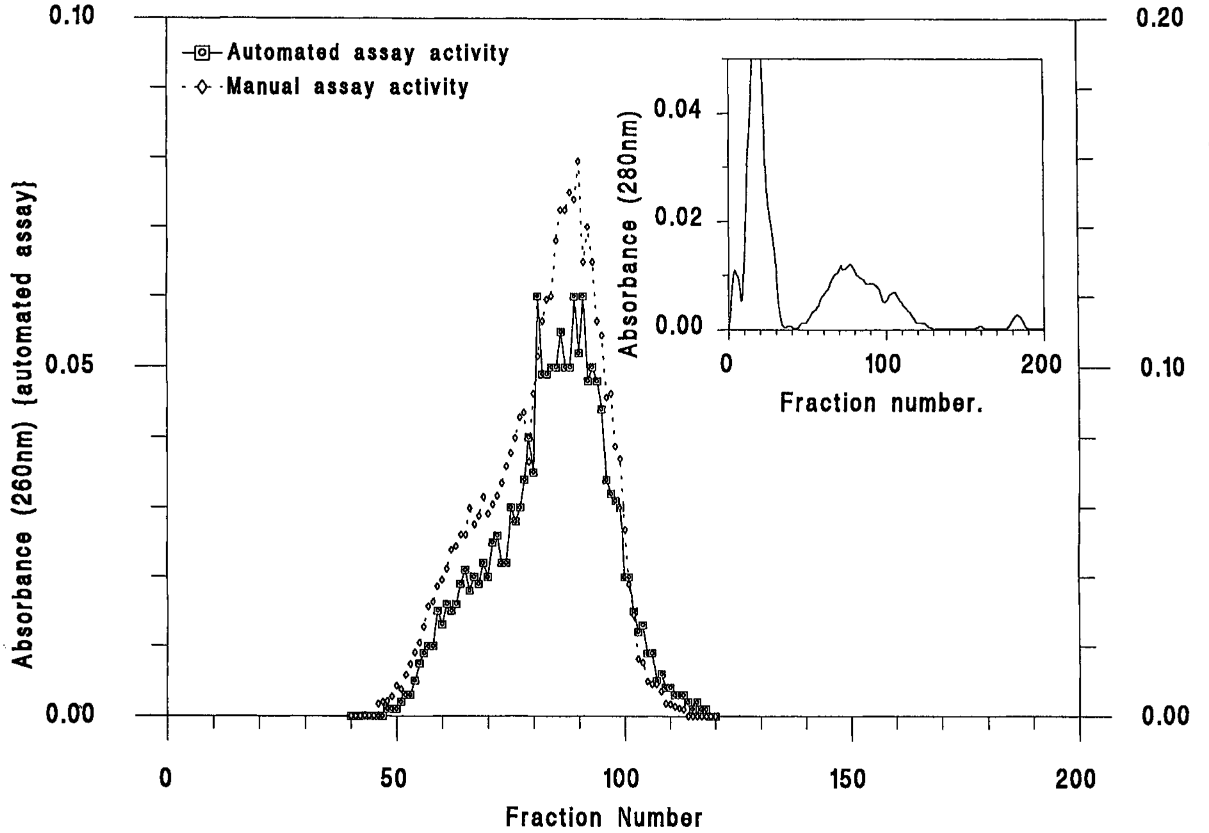

*Figure 3. Comparative activity profiles for both the manual and automated assay of chromatographic fractions from Gel Filtration on Bio-Gel P-100 using pH 7·4 phosphate buffer (0·05 M). Automated assay results are presented as a solid line and manual results as a broken line. The inset shows the column elution profile as monitored for protein absorption at 280 nm.*

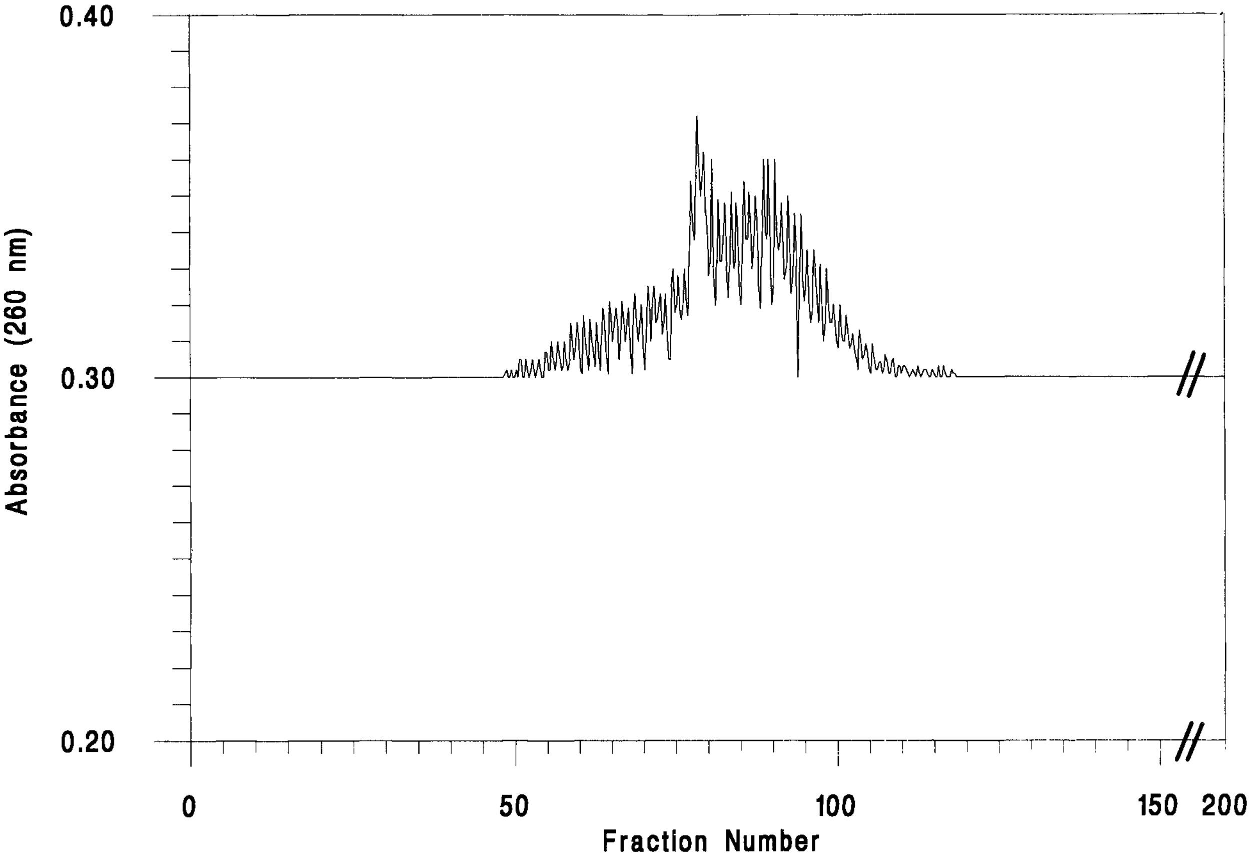

*Figure 4. Typical AutoAnalyser output for analysis of chromatographic fractions from gel filtration on Bio-Gel P-100, with pH 7·4 phosphate buffer (0·05 M) mobile phase, using the AutoAnalyser schematic shown in figure 1.*

heating coil. This was of a greater length than the first and was placed in the substrate feed line to pre-heat the substrate DNA to 30 °C. This ensured that the smaller secondary coil was sufficient to keep the reaction mixture at its optimum temperature.

As a consequence of introducing the dialysis units it became necessary to introduce air segmentation into the sample line. Without segmentation there was considerable mixing of different samples as they entered the dialysers resulting in 'bleed-over' of activity when the samples were read. The addition of the air bubbles prevented this. After passing the dialyser units, the sample line must merge with the main substrate flow, where the presence of the smaller, secondary, air bubbles of the sample stream between the main segmenting bubbles was thought to be reducing the efficiency of the mixing stage which followed. In order to overcome this, the 'h3' connector (Part No. 116-0211) was orientated so that it was in the vertical plane at an angle of approximately 60° from the horizontal (see figure 2). The main segmenting bubbles were introduced into the substrate stream from above and the sample stream merges from below. With such an arrangement, the sample stream air bubbles rise as they enter the substrate stream and 'collide' with the main air bubbles at their point of introduction. The force of this collision causes the air bubbles to coalesce, hence removing the mixing problem.

A typical elution profile of the gel filtration of Varidase using Bio-Gel P100 monitored to indicate the presence of proteins is presented in figure 3. Also shown in figure 3 are the fractions which contain deoxyribonuclease activity. It can be seen that the activity is contained, within approximately the fractions 50–105, but the maximum activity does not coincide with the indicated maximum protein concentration.

The nuclease activity can be considered as being partially resolved into at least two separate regions. Both the manual and automatically accumulated data generated profiles whose overall shapes are in excellent agreement. It is to be expected that the actual magnitude of the peaks are not coincident, as the units of activity from the manual and automated methods are not the same. The data output from the automated system (figure 4) appears as a succession of peaks above the level background absorbance of the DNA substrate solution. The heights of these peaks, representing the increase in absorbance after a constant elapsed time of nearly 3 min, are proportional to the nuclease activity. The manual activity is calculated from the initial linear absorbance increase with time following mixing of the enzyme and substrate.

The nuclease activity monitored from the elution of Varidase from a Cellex-D column using a 0·05–1·0 M salt gradient is interesting in two respects. First, it shows that the nuclease activity is confined to a single protein peak, as measured by absorbance at 280 nm. Second, the

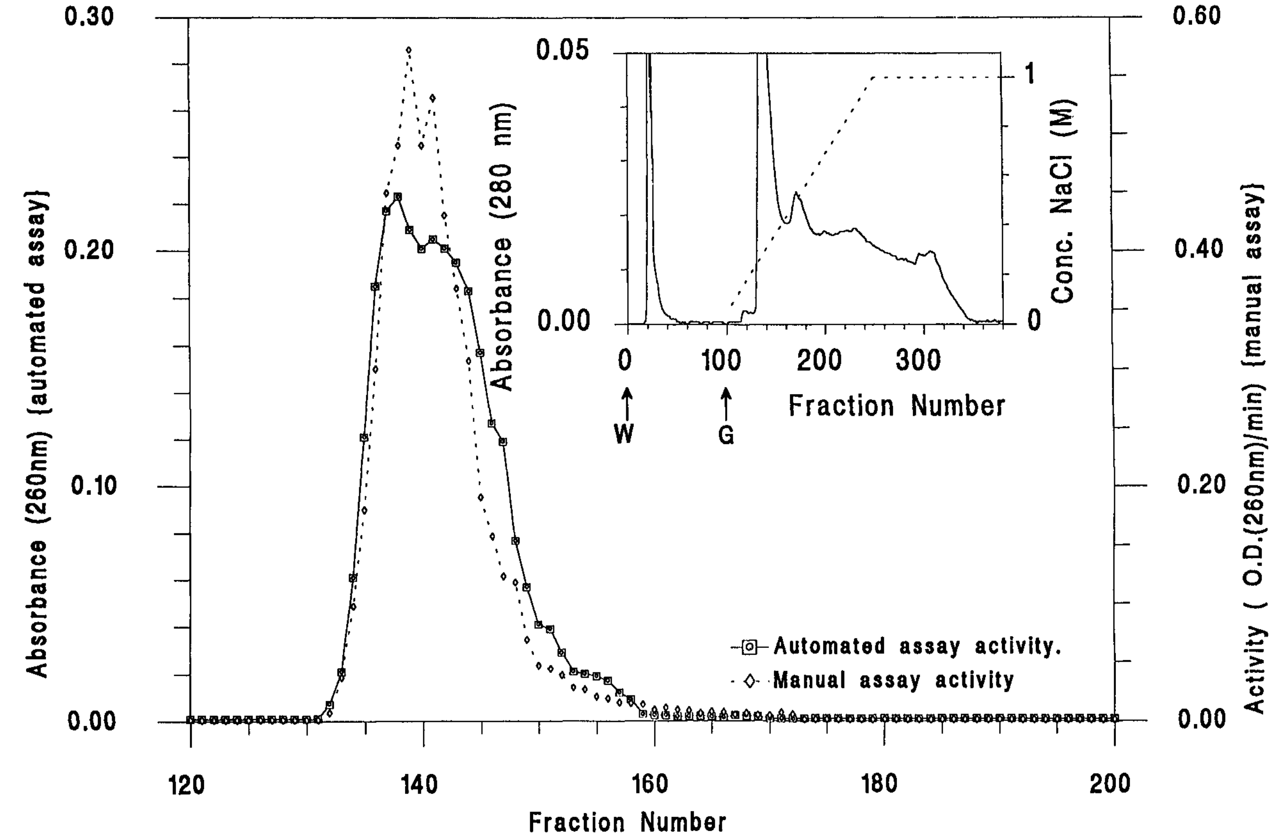

*Figure 5. Comparative activity profiles for both the manual and automated assay of chromatographic fractions from ion-exchange. Automated assay results are presented as a solid line and manual results as a broken line. The inset shows the column elution profile as monitored for protein absorption at 280 nm with the sodium chloride elution gradient shown in overlay as a broken line. 'W' indicates the point at which the phosphate buffer wash was started, 'G' indicates the point at which the sodium chloride gradient was applied.*

automated and manually determined activity profiles show a slight, but important, difference. Both profiles show a resolution into two activity regions within the overall protein peak. However, at fraction 145 the manual and automated curves cross over. From then until fraction 160 both curves follow a similar variable outline, the manually determined activity being of lower magnitude than the automatically determined one. In the higher numbered fractions, those activities determined manually contain increasing amounts of salt as no attempt was made to remove any constituents of the eluted column fractions before testing their activity. In the automated system the salt had been dialysed out. This registered change in activity is therefore entirely expected and is in agreement with previous observations [4], which have demonstrated a decrease in nuclease activity with increasing salt concentration. Again, due to the distinction in activity units, the manually determined activity is registered as being generally higher than that from the automated system. The slight variations in the two profiles between fractions 160–166 are due to the lower sensitivity of the automated system compared to the manual one.

### Conclusions

It has been demonstrated that the Technicon AutoAnalyser can be adapted to perform automated assays of deoxyribonuclease activity by monitoring the change in absorbance at 260 nm brought about by the breakdown of double-stranded deoxyribonucleic acid. The automated assay schematic can be applied to chromatographic fractions eluted with buffers of both constant and variable sodium chloride content. Comparison of the automated profiles with those determined manually (see figure 5) show complete agreement of relative activities where there is elution in constant buffer conditions. However, there are discernible differences when the elution takes place with increasing salt concentration. The automated system gives a more realistic profile of the relative deoxyribonuclease activity of the various fractions as the inhibitory effects of the sodium chloride have been removed.

Although this assay should be adaptable to measure most deoxyribonuclease activities, it does rely on the nuclease digesting double-stranded DNA into small fragments or nucleotides. Restriction endonucleases which cut DNA only at a number of distinct, sequence specific, sites will produce very little absorbance change at 260 nm and would not be amenable to the automated assay in its present form.

### Acknowledgements

We wish to thank Mr R. E. Hone for his advice and assistance during the assembly of the Technicon AutoAnalyser schematic.

### References

1. Hawkins, S. R., *Methods of Producing Streptokinase and Streptodornase. US Patent No. 2,702,781*, Issued February 22 1955.
2. Tillet, W. S., *Annals New York Academy of Sciences*, **68** (1957), 151.
3. Tillet, W. S., *Harvey Lectures*, **45** (1949–50), 1149.
4. Hill, S. S., PhD Thesis (CNAA) Portsmouth Polytechnic, (1990).
5. Price, P. A., *Journal of Biological Chemistry*, **247** (1972), 2895.
6. Junowicz, E. and Spencer, J. H., *Biochemica Biophysica Acta*, **312** (1973), 72.
7. Wannamaker, L. W. and Yasmineh, W., *J. Experimental Medicine*, **126** (1967), 475.
8. Wannamaker, L. W., Hayes, B. and Yasmineh, W., *Journal of Experimental Medicine*, **126** (1967), 497.
9. Melgar, E. and Goldthwait, D. A., *Journal of Biological Chemistry*, **243** (1968), 4409.
10. Campbell, V. W. and Jackson, D. A., *Journal of Biological Chemistry*, **255**(8) (1980), 3726.

